# Curare - A Curative Poison: A Scientometric Analysis

**DOI:** 10.1371/journal.pone.0112026

**Published:** 2014-11-19

**Authors:** Jil Carl, Mario Schwarzer, Doris Klingelhoefer, Daniela Ohlendorf, David A. Groneberg

**Affiliations:** Institute for Occupational Medicine, Social Medicine and Environmental Medicine, Goethe University, Frankfurt, Germany; Cincinnati Childrens Hospital Medical Center, United States of America

## Abstract

**Introduction:**

Curare is one of the best-examined neurotoxins of the world, which has empirically been used for centuries by American Indigenes. Research on curare has been performed much later, a global scientometric analysis on curare research or its derivates does not yet exist. This bibliometric analysis is part of the global NewQis-project and should illuminate both toxic and historic issues of research on curare.

**Methods:**

The ISI Web of Science was searched for data covering 1900 to 2013 using a term which included as many original articles on curare as possible. 3,867 articles were found and analyzed for common bibliometric items such as the number of citations, language of the articles or the (modified) Hirsch-Index (h-index). Results are illustrated utilizing modern density equalizing map projections (DEMP) or beam diagrams.

**Results:**

Most publications were located in North America and Europe. The USA has the highest number of publications as well as the highest h-index. The number of publications overall rose until the late 1990s and later decreased. Furthermore, sudden increases of research activity are ascribable to historic events, like the first use of curare as muscle relaxant during surgery.

**Discussion:**

This scientometric analysis of curare research reflects several tendencies as previously seen in other bibliometric investigations, i.e. the scientific quality standard of North America and Europe. Research on curare decreased however, due to the declining attention towards this muscle relaxant. This work exemplifies also how scientometric methods can be used to illuminate historic circumstances immediately stimulating scientific research.

## Introduction

Curare is used for centuries by humans, and its toxic patho-mechanism [1] has been meticulously examined. This insidious neurotoxin has killed many humans and animals over the centuries.

At first, curare was known as “arrow poison”. The indigenes of America used it over centuries for hunting and produced this poison, by boiling diverse plants, e.g. Chondrodendron tomentosum, Menispermaceae or Strychnos, according to traditional recipes. The resulting paste was applied to arrowheads. Prey animals, struck by the poisoned weapons, died within a few minutes. Hence, it was just a question of time, until the underlying molecular mechanism aroused interest in European scientists as well as physicians [2]–[5].

But how did curare find its way out of the jungle onto European laboratory benches? In the 15^th^ century – the era of the conquerors – Christopher Columbus and his crew on behalf of the Spanish crown set sail to search for a western route to India, crossing the Atlantic Ocean. Leaving the Canary Islands behind, they made landfall believing they found a seaway to East Asia. Realizing it as a new continent, it was called the “New World”; the rest is history.

During the local expeditions, the crew came under fire from natives. Poisoned arrows hit two members of the crew. Both men quickly died in agony in spite of relatively small wounds. They discovered a brown paste on the arrowheads and assumed poison on the arrows, which hit their friends [3], [4]. Columbus reported of this case in Europe, where it was published by Peter Martyr D'Anghiera in his book “De Orbe Novo” [6].

This was the time scientific research on curare started. Curare was prepared and distributed but still was a long process of experiments and failures until the mysteries of the poison out of the jungle of South America could be decoded. Walter Raleigh, Charles Marie de la Condamine and Claude Bernard became known for their investigations on curare and made eminent achievements on this scientific field and promoted the interest of anesthesiologists towards this toxin [2], [3], [5].

Especially Alexander von Humboldt played an important role in the history of curare. In 1800 he started a journey towards Esmeralda with his companion Aimé Bonpland and got to know the chemist of the village. They were the first Europeans to be shown the tribal traditional production of curare. Humboldt accurately documented which instruments were necessary and how they were used, one of the best stories of medical history [7].

The two men trusted the experience and knowledge of the old Indian. He confirmed that neither the steam of hot curare preparations are toxic nor its ingestion. Humboldt decided to drink the potion and proved that only parenteral contact to curare is deadly [7].

Another hundred years were necessary to decrypt the curare secrets.

In the 20^th^ century the molecular mechanism of curare as a competitive antagonist of nicotinergic neuromuscular synaptic junctions was finally elucidated. This non-depolarizing muscle relaxant, once in the circulation, quickly leads to paralysis including respiratory paralysis. Prey animals could not run away once they were hit by an arrow, and died within a short period of time [8].

Curare wasn't used clinically for muscle relaxation until 1942. Although patients were anesthesized for surgery -the dentist William Morton successfully permormed the first general anesthesia on October 16^th^ 1846 [9] - the problem of muscle contractions was not solved.

Since curare could cause complete immobility of the patient and allowed the surgeon to operate under ideal conditions, it gained scientific popularity since the end of the 19^th^ century.

The first clinical use of curare as muscle relaxant during an operation is reported on January 23^rd^ 1942. The anesthesiologist Harold Griffith injected a synthetic preparation of curare to a young man before appendectomy. The era of muscle relaxants in surgery had started [10].

With the on-going rapid development of medical science new derivates could be synthesized with better pharmaceutical characteristics, so the original curare from Amazonia lost its relevance in modern medicine. Still a lot of research was necessary to develop the modern drugs in current use.

## Aim of this study

No scientometric analysis of the research on curare or its derivates exists. This study focuses on original articles of curare research since 1900. The results illustrate the development of scientific research activity of this neurotoxin over the decades; on the other hand we address the shift in research focus. Classical bibliometric parameters are therefore used like the total number of published items for countries, authors, number of citations or the citation rate using novel visualizing methods like "density equalizing map projections". This work is part of the global NewQis-project [11], testing methods towards their possible application for historical analyses.

This scientometric analysis elucidates whether the success story of curare can be ascribed to single scientists like Alexander Humboldt alone or further factors being additionally accountable. This analysis should also shed light on the research activity in this insidious but bygone tropical cause of death.

## Methods

### Data source

The analysis is based on the NewQis-platform [11] using records provided by the database Web of Science (WoS; Thomson Reuters, Philadelphia, USA), since it includes representative journals with impact factors listed. A qualitative analysis of the citation report function is also feasible.

In order to include all published items, the database was searched using the following search term limited to the title: “*curare OR *curarin OR *curonium OR *curium OR *curarization” NOT (curium OR *transcurium OR *mercurium)”. This strategy should focuse on curare; the chemical elements curium, "mercury" and transuranium elements were excluded. In a second step the results were refined for the document type article excluding congress abstracts. The search was done on December 31^st^, 2013.

### Evaluation criteria

#### General publication characteristics

The collected bibliometric data was analyzed for the number of publications and citations as previously described [12]. Briefly:

#### Geographical distribution

For the geographical distribution the items "number of publication" as well as the "modified Hirsch-Index" (h-index) were calculated and the results illustrated by Density Equalizing Map Projections (DEMPs), which were developed by Gastner and Newman [13]. The h-index was calculated from the number of publications and the citation rates. A country with an h-index of x has published x papers each being cited in other papers at least x times [14]. This index reflects both the number and the impact of publications making it particularly suitable as a bibliometric scale for the qualitative analysis of scientific research [15].

While based on conventional geographic maps, a DEMP displays countries or continent size proportional to a certain attribute, e.g. pulbication number or h-index. Using this procedure, complex data can be visualized in a very accessible and plausible way. Ultimately the diffusion-based DEMP is the representation of country sizes by a given factor as distorted geographical map [13], [16].

#### Collaborations

nternational collaborations were analyzed by using the listed addresses. Countries which more than five international collaborations were illustrated by a beam diagram, the width of the beams is proportional to the volume of cooperation.

#### Journals

A journal analysis was performed similar to the author analysis. We identified the journals with the highest numbers of articles and citation rates and showed the number of citations per article for the top 15. The citation rate was used as a qualitative marker comparable to the well known impact factor (IF), which corresponds to a journal's citation rate of the last two years.

#### Subject areas

The original WoS content categories (subject areas), which include articles regarding curare, were analyzed in decade periods to illustrate time shifts from 1900 to present.

#### Languages

The shift of language use from 1900 up to now was illustrated analogously to the subject areas.

## Results

### Publications

**Figure 1:**
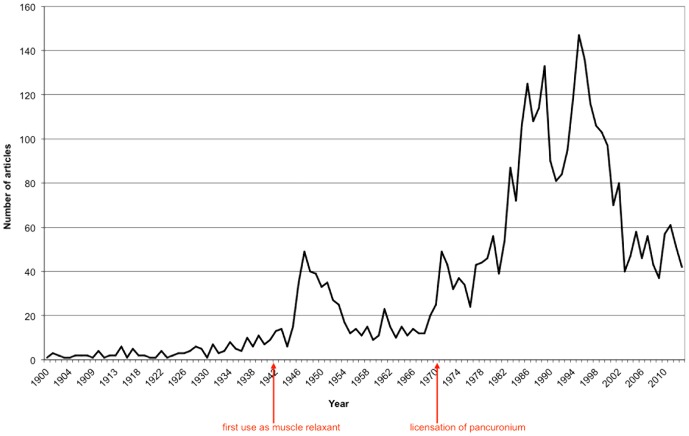
Articles by years.

Overall, 3,867 articles regarding curare are found in WoS. The development of the number of publications per year since 1900 is illustrated in Fig. 1. From 1900 to 1940 the publication rate remains low at about 12 articles per year. This value rises significantly in 1942 and peaks with 47 articles in 1947 coinciding with the clinical use of curare as muscle relaxant. Between 1955 and 1965 the publication rate decreases again to appr. 15 articles per year. Since 1966 it rises again with one peak in 1972, and one in 1989 to 1995. After 1995, the year with the most publications, the publication rate decreased to approximately 50 articles/year.

### Citations

Until 1930 curare articles were rarely cited. Since then, citations increased constantly, with the first peak in 1950 (646 citations). The number of citations per year reached its maximum in 1983 (2487 citations) and decreased continuously afterwards. The number of citations closely followed the number of publications.

### Geographic analysis

**Figure 2:**
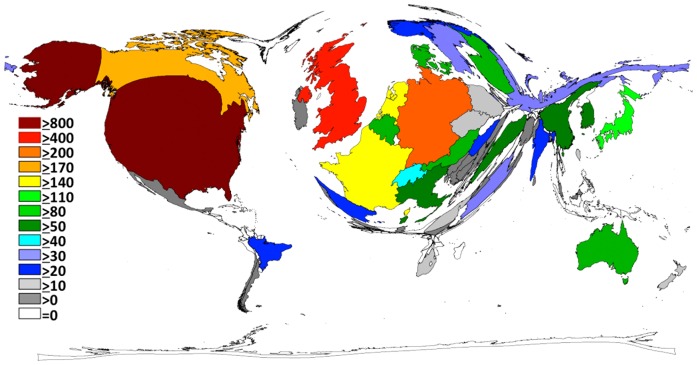
Global distribution of the number of published items.

The number of publications for each country is illustrated in Fig. 2. The USA has the highest scientific output worldwide with 897 articles followed by the United Kingdom (476 articles), Germany (233 articles), Canada (177), France (162 articles), the Netherlands (152 articles) and Japan (118 articles).

**Figure 3:**
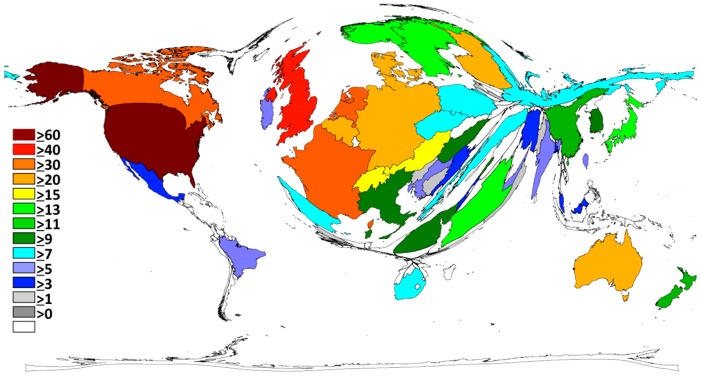
Global distribution of the modified h-index.

The ranking of the number of publications using the h-index changes this order (Fig. 3). Whereas the USA is leading with an h-index of 62 followed by the UK (45), Netherlands achieved an h-index of 34, Canada (32) and France (31) following closely. Overall, North America and Europe are the most prolific and the most influential countries in curare research.

### International collaborations

**Figure 4:**
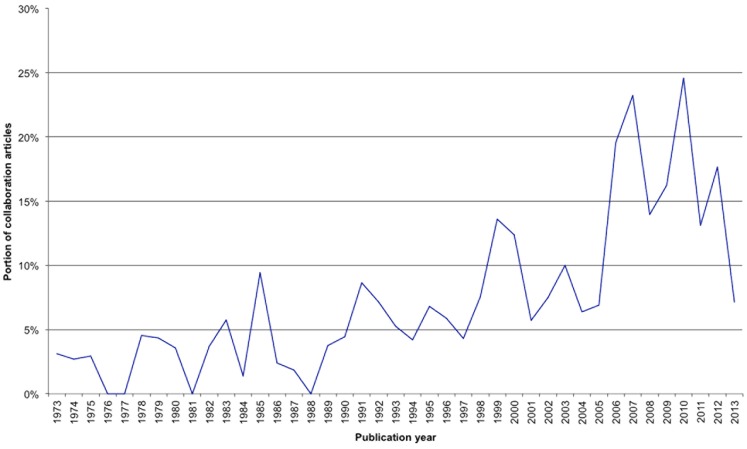
Collaborative articles in percent of all published articles by years.

The percentage of articles originating from international collaborations of at least two countries increased continuously (Fig. 4).

**Figure 5:**
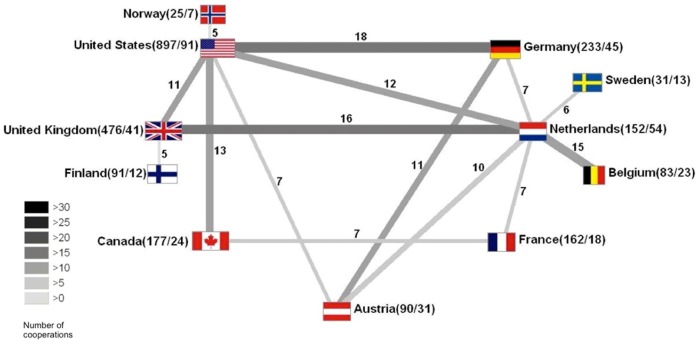
Global collaborations - Each country is represented by its flag, while the first number in brackets represents the total volume of national publications, the second one represents the number of cooperation articles of the specific country, the number above the beam represents the number of collaborative articles.

The collaborations are visualized by a beam diagram in Fig. 5. The USA (91 studies with international collaborations) in general has the most collaborative articles overall, but the Netherlands (54) has more different collaboration partners. USA and Germany (45) share the strongest partnership with 18 articles. Strongly internationally cooperating countries also are the United Kingdom (41), Canada (24) and Sweden (13).

### Journals

**Figure 6:**
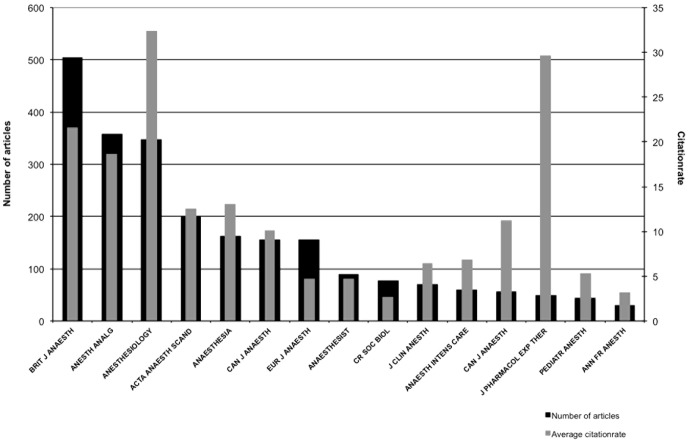
Top 15 journals by articles.

The 15 journals with the largest number of articles on curare and its derivatives are shown in Fig. 6. The "British Journal of Anaesthesia" (504 articles) has published the highest number followed by "Anesthesia & Analgesia" (357 articles) and "Anesthesiology" (347 articles).

The "Canadian Journal for Anaesthesia" (155 articles) was formerly known as "Journal canadien d'anesthésie" (55 articles), these journals share the same abbreviation (Can J Anaesth). The top 15 journals are published in the USA (Anaest Analg, Anesthesiology, J Clin Anesth, J Pharmacol Exp Ther), the United Kingdom (Brit J Anaesth, Anaesthesiology, Pediatr Anesth), France (CR Soc Biol, Ann Fr Anesth), Canada (Can J Anaest), Switzerland (Eur J Anaesth), Australia (Anaesth Intens Care) and Germany (Anaesthesist).

The citation rate (Fig. 6) does not correlate to the number of published articles. "Anesthesiology" has the highest citation rate with 32 citations per article, followed by the "Journal of Pharmacology & Experimental Therapeutics" (30), which published only 48 articles on curare.

### Subject Areas

**Figure 7:**
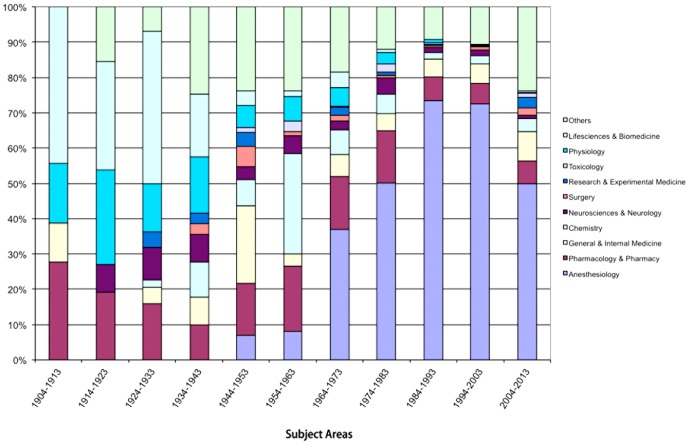
Shift of subject areas by decades.

The content categories (subject areas) have connotatively changed over the decades (Fig. 7). "Life Sciences & Biomedicine" is the subject area with the most articles published on curare from 1904 to 1943. In the 1950s and 1960s the main publishing subject area has been "General & Internal Medicine", since then most articles have been published in the subject area "Anesthesiology" Articles in "Pharmacology & Pharmacy" are found in all periods other subject areas like "Physiology" lose their influence.

### Languages

**Figure 8:**
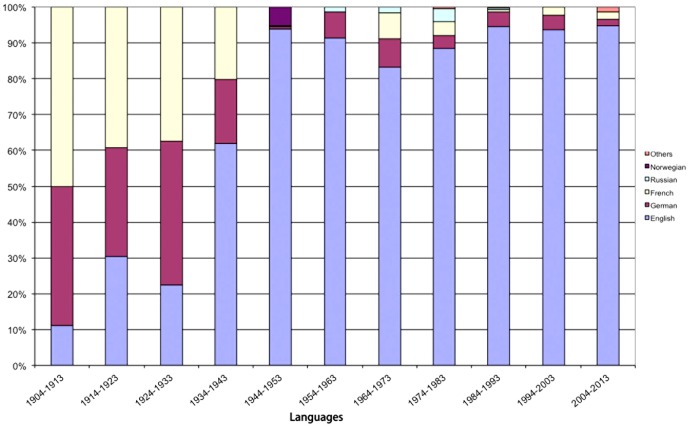
Shift of languages by decades.

The development of the article languages is illustrated analogously to the subject areas (Fig. 8). Until 1933 the dominating languages were French and German. Afterwards English became the most relevant language for scientific research. Currently (2004–2013) more than 90% of the articles on curare are written in English.

## Discussion

This study evaluates and illustrates the scientific research of curare and its derivates from 1900 to 2013. Methods and techniques which have been developed for the NewQis-Project [11] also are tested for their suitability regarding historical examinations.

Some bibliometric parameters of curare research are identical to other topics [17], [18], i.e. the rising number of articles, especially since the early 1980s. Increasing publication output may be ascribed to modern technologies as the Internet, which provides additional scientific databases, publishing scientific results and accessing many different journals and publications has become much easier. It also has to be noted that monetary incentives often support a high publication output. Hence scientists worldwide publish an increasing number of articles, often as “smallest publishable unit”. Consequently, the numbers of articles rises listed in the global databases.

This analysis on curare exemplifies some additional features, which are exclusive for the research on curare. For example, the first two peaks of publications per year (Fig. 1) can be allocated directly to historical events. The first peak around 1947 is three years after the first clinical use of a curare derivate as muscle-relaxant during an operation. The second specific rise in articles is appr. 1972 closely following the clinical introduction of new curare derivates like *pancuronium* [19]. These events illustrate the reaction of medical research to new developments.

In addition, the number of articles since 2004 decreased to 40 - 60 articles per year, a comparatively low level corresponding to the output of the 1970s, and is a rare development in medical research, compared to other expanding medical fields (e.g. [20], [21]). It can be assumed that the clinical use refocused to other muscle relaxants like depolarizing agents. Curare and its derivates are the oldest muscle relaxants in use, so the strong interest in these substances seems to vane slightly.

The distribution of curare research between countries is completely unequal (Fig. 2 and Fig. 3). The number of published articles as a quantitative parameter and the modified country-specific h-index as a qualitative parameter indicate that North America, i.e. USA and Canada, has the strongest qualitative and quantitative impact on the knowledge of curare. The second major area of research is Europe in general. The large difference between North America and Europe, as well as the rest of the world, disappears slightly when looking at the h-index as a quality parameter. The h-index flattens the distortion in quantity (number of articles).

The focus of the research towards curare has changed dramatically since 1900 (Fig. 7). It can easily be seen that the early publications on curare deal with its physiologic effect on animals or humans. Later, the major focus is research in the use of curare as muscle relaxant in anesthesia. Contemporary research focuses on curare as a drug. This may explain the dominance of the Western world (North America, Europe and Australia), in which large pharmaceutical companies strongly support drug research.

This study began with the question whether the success of decrypting the mysterious jungle poison and the development of potent muscle relaxants can be ascribed to a single person like Humboldt or his friend Bonpland or to a group effort. It seems that individual persons have given important impulses, e.g. Harold Griffith who was the first surgeon using a curare derivate during an operation, but the biggest contributions to our knowledge are ascribed to larger groups in international collaborations between scientists. These collaborations seem to become ever more productive over the last few decades (Fig. 4). It has to be assumed that international collaborations will even be extended in the future with the Netherlands as an example. They have a relative low output level of high quality publications as seen by its h-index. It can be assumed that this effect is due to the large number of collaborations as well as the high percentage of collaborative articles (see Fig. 5). In comparison to the USA (91 collaborations, 897 articles), the Netherlands have 30% collaboration articles (54/152). It may be stated that internationally collaborating countries, institutes or scientists promote their scientific impact by all partners and therefore improve their output rate including financial aspects, too.

Thus the cooperation of scientists from different countries strengthened curare research as indicated by the increasing number of collaborative articles and the high article quality represented by a high modified h-index. Today, the impact of single scientists like Humboldt seems less obvious.

The methods and techniques developed within the NewQis-project are suitable for scientometric examinations. This investigation both shows historically interesting aspects of the curare research as well as prognostic research aspects in general and specifically medical sciences, i.e. the outstanding importance of international collaborations. This global analysis also shows that even deadly substances from the jungle such as curare may gain importance in Western research and be developed into health products.

## Supporting Information

Rawdata S1 (TXT)

Rawdata S2 (TXT)

Rawdata S3 (TXT)

Rawdata S4 (TXT)

Rawdata S5 (TXT)

Rawdata S6 (TXT)

Rawdata S7 (TXT)

Rawdata S8 (TXT)

Rawdata S9 (TXT)
